# Trade-Offs between Predation Risk and Growth Benefits in the Copepod *Eurytemora affinis* with Contrasting Pigmentation

**DOI:** 10.1371/journal.pone.0071385

**Published:** 2013-08-07

**Authors:** Elena Gorokhova, Maiju Lehtiniemi, Nisha H. Motwani

**Affiliations:** 1 Department of Applied Environmental Science, Stockholm University, Stockholm, Sweden; 2 Finnish Environment Institute, Marine Research Center, Helsinki, Finland; 3 Department of Ecology, Environment and Plant Sciences, Stockholm University, Stockholm, Sweden; University of Shiga Prefecture, Japan

## Abstract

Intraspecific variation in body pigmentation is an ecologically and evolutionary important trait; however, the pigmentation related trade-offs in marine zooplankton are poorly understood. We tested the effects of intrapopulation phenotypic variation in the pigmentation of the copepod *Eurytemora affinis* on predation risk, foraging, growth, metabolic activity and antioxidant capacity. Using pigmented and unpigmented specimens, we compared (1) predation and selectivity by the invertebrate predator *Cercopagis pengoi*, (2) feeding activity of the copepods measured as grazing rate in experiments and gut fluorescence *in situ*, (3) metabolic activity assayed as RNA:DNA ratio in both experimental and field-collected copepods, (4) reproductive output estimated as egg ratio in the population, and (5) total antioxidant capacity. Moreover, mitochondrial DNA (mtDNA) COI gene variation was analysed. The pigmented individuals were at higher predation risk as evidenced by significantly higher predation rate by *C. pengoi* on pigmented individuals and positive selection by the predator fed pigmented and unpigmented copepods in a mixture. However, the antioxidant capacity, RNA:DNA and egg ratio values were significantly higher in the pigmented copepods, whereas neither feeding rate nor gut fluorescence differed between the pigmented and unpigmented copepods. The phenotypic variation in pigmentation was not associated with any specific mtDNA genotype. Together, these results support the metabolic stimulation hypothesis to explain variation in *E. affinis* pigmentation, which translates into beneficial increase in growth via enhanced metabolism and antioxidant protective capacity, together with disadvantageous increase in predation risk. We also suggest an alternative mechanism for the metabolic stimulation via elevated antioxidant levels as a primary means of increasing metabolism without the increase in heat absorbance. The observed trade-offs are relevant to evolutionary mechanisms underlying plasticity and adaptation and have the capacity to modify strength of complex trophic interactions.

## Introduction

Variation in pigmentation among individuals, populations and species has been associated with various adaptations to predation, ultraviolet radiation (UVR) and diet, but the ultimate mechanisms and trade-offs regulating the observed patterns in the field are still unclear. Studies on pigment variation and its adaptive function in zooplankton have been focused on carotenoids in calanoid copepods [1], [2] and melanisation of daphniids [3], [4], [5]. Most of these studies attributed pigmentation variability to the trade-offs between protection from UVR and predation [6], [7], with weak pigmentation correlating with lower predation pressure but also with lower UVR tolerance [4]. Deeply colored populations are usually found at high-latitude or high-altitude lakes, but pigmentation is also observed in temperate regions [7], [8]. In temperate marine zooplankton, the evidence for the linkage between pigmentation and UVR tolerance is, however, less compelling. For example, the pigmentation does not appear to play a role in UVR tolerance of marine copepods [9], whereas the opposite was observed in crab larvae [10].

In addition to the photoprotective function of pigments, a metabolic stimulation hypothesis has been advocated to explain the adaptive value of pigmentation by providing warmth through absorption of solar radiation [11], [12]. Any increase in body temperature due to energy absorbance by body pigments under light conditions would thus have primarily positive effects, such as increased enzyme activities, metabolic rates and, ultimately, growth. Laboratory experiments have shown that under sunlight, respiration rate increased in pigmented copepods but not in those lacking visible pigmentation [11], [12], and that average pigmentation intensity was inversely related to temperature [6], [12]. This has important implications for zooplankton performing diel vertical migrations (DVM) as demographic disadvantage to zooplankton that spend a daytime at deeper and cooler waters may actually be similar to or greater than the lethal effects of predation [13]. In fishless lakes, pigmented zooplankters remain in productive near-surface waters during the day and have higher reproduction rates [14]. Therefore, by relaxing DVM and increasing pigmentation, a zooplankter can minimize photodamage, enjoy high food availability and reproductive output in the illuminated trophogenic zone, but at the cost of predation by visual planktivores. Although, fish are commonly considered as the only visual predators in aquatic ecosystems, there are marine and freshwater invertebrates capable of visual predation, such as mysids [15] and onychopod cladocerans [13], [16]. The invasive onychopods *Cercopagis pengoi* and *Bythotrephes longimanus* have been implicated in the reduction of zooplankton diversity and abundance in the Laurentian Great Lakes [17] and the Baltic Sea [18] as well as in the forcing zooplankton to migrate below thermocline during daytime [13], [18]. By studying intraspecific and intrapopulation variability in pigmentation in relation to growth and vulnerability to predation, we can better understand mechanisms of these trade-offs in specific populations and environmental conditions.

Many zooplankton pigments are important quenching agents and antioxidants that neutralize reactive oxygen species; they also have other biological functions, such as regulatory effects on cell signaling [19]. The changes in the antioxidative status may thus translate to fitness responses, and increase in investment in the antioxidant system can come at a cost of an investment elsewhere, e. g., it may shorten lifespan, decrease growth and reproductive output, weaken immune system, etc. [19], [20]. However, in different species, elevated growth has been linked with both a reduction and an increase in antioxidants [20]. This is related to a complexity of antioxidant systems and interactions amongst various antioxidants, implying that any specific antioxidative compound may fail to indicate the overall defense state. Thus, responses of individual antioxidants are generally more difficult to interpret than those of overall antioxidant capacity when testing specific relationships between antioxidants, metabolic activity and growth, in order to understand mechanisms of trade-offs.

As far as we know, no studies have addressed pigmentation-mediated trade-offs between fitness parameters and vulnerability to predation by invertebrate predators in marine zooplankton. Moreover, whereas genetic basis and developmental mechanisms generating the diversity of pigmentation and color patterns are well appreciated in vertebrates [21], much less is understood regarding genetic versus environmental influence on pigment variation in invertebrate species under various selection pressures acting on pigmentation-related traits. In *Daphnia*, both genetic and environmental effects were found to influence pigmentation [14], [22], with differences in melanisation among species, clones and mitochondrial DNA (mtDNA) haplotypes [23], [24]. Similarly, a genetic divergence in mtDNA was found between differently pigmented but otherwise morphologically identical varieties in shrimps [25] and ascidians [26].

In our fieldwork in different areas of the Baltic Sea, we were puzzled by variability in pigmentation in the calanoid copepod *Eurytemora affinis* Poppe, raising questions about the factors that influence pigmentation in this species. This copepod is one of the few dominant mesozooplankton species in the northern Baltic and a preferred prey for the dominant zooplanktivores [27] as well as for *C. pengoi* [18]. Interestingly, a recent inventory using mtDNA, revealed a high diversity in the Baltic lineages of *E. affinis* [28]. This species has been suggested to actually comprise a number of cryptic species [29], two of which were recently reported from the Baltic Sea [30], [31]. Given the observed genetic variation in the Baltic *Eurytemora*, differences in pigmentation between the cryptic species and association between pigmentation and certain mtDNA haplotypes in *E. affinis* are possible.

In this study, we investigated whether the observed variation in pigmentation is related to interspecific variation between *Eurytemora affinis* and a morphologically similar species *E. carolleeae* recently reported from the Gulf of Finland [31]. When the presence of only original Baltic lineage of *E. affinis* in our collections was confirmed, we examined the intrapopulation variation of the mitochondrial cytochrome oxidase *c* subunit 1 (COI) gene in relation to pigmentation, and tested effects of this pigmentation on the predation and selectivity by *C. pengoi* and on the variations in antioxidant capacity, feeding, metabolic activity and reproduction of the copepods.

## Methods

### Ethics Statement

The sampling was conducted within national Finnish monitoring in the Gulf of Finland and no specific permissions were required for the sampling locations of this study. Also, we did not require ethical approval to conduct this study as we did not handle or collect animals considered in any animal welfare regulations and no endangered or protected species were involved in the samplings or the experiments.

### Defining Pigmented and Unpigmented Forms

The term pigmentation is used here to describe the presence of a dark pigment in the exoskeleton of *E. affinis*. There was a clear distinction between pigmented and unpigmented individuals, with the latter appearing opaque or transparent in transmitted light, without visible dark coloration of any specific area, apart from the eye, ovaries and egg sacks (in females). By contrast, the pigmented individuals had dark brown coloration on either one side of the body or on the both sides; the pigmentation was particularly strong on antennae, ventro-lateral part of cephalothorax and *furcal rami* (Figure S1).

### Sampling Methods, Locations and Sample Preparation

The experimental animals were collected and the experiments were conducted on board R/V ‘*Aranda*’ (SYKE, Finland) in August 2011 in the eastern and central Gulf of Finland, the northern Baltic Sea (Table 1). *Eurytemora affinis* and *Cercopagis pengoi* were collected from 20 m to the surface, using a WP-2 plankton net (mesh size 100 µm) equipped with a cod end. The samples were used to obtain animals for the experiments and to preserve them for different measurements. For predation and grazing experiments, *E. affinis* individuals with contrasting pigmentation were sorted with a pipette under a dissecting microscope and kept in groups of 10 individuals in 0.2 µm-filtered seawater (FSW) in 0.1 L vials. For the predation experiments, *C. pengoi* were carefully picked under a dissecting microscope with forceps and wide-mouth pipettes and transferred individually to FSW in 0.1 L vials; only actively swimming individuals were used in the experiments. All specimens were kept at 16°C approximating the temperature in the mixing layer until the experiments commenced. The remaining zooplankton collections were treated as follows. The water was removed as much as possible with a filter paper and the samples were preserved by: (1) freezing at −80°C for measurements of gut fluorescence and antioxidant capacity; and (2) adding RNA*later* 10∶1 (v/v) for genetic analysis and measurements of nucleic acid concentrations; these samples were also used for microscopic analysis to estimate sex- and age-related difference in pigmentation and to calculate egg ratio.

**Table 1:** Details on sampling dates, locations, sample usage and preservation.

Date	Station	Latitude	Longitude	Species	Experiments and preservation for further analyses
8 Aug	LL6	N59°53.01	E025°11.81	*E. affinis*	Exp 1 &2, RNA*later*
8 Aug	LL5	N59°55.01	E25°35.82	*E. affinis*	Exp 1 &2
9 Aug	LL3a	N 60°04.03	E 26°20.80	*E. affinis*, *C. pengoi*	Exp 1 &2, RNA*later*, –80°C
9 Aug	XVI	N60°15.00	E27°14.82	*E. affinis*, *C. pengoi*	Exp 1 &2, Exp 3
9 Aug	XIV3	N60°12.19	E26°11.57	*E. affinis*, *C. pengoi*	Exp 1 &2, RNA*later*, –80°C
10 Aug	GF1	N59°42.30	E024°40.93	*E. affinis*, *C. pengoi*	Exp 1 &2, RNA*later*, –80°C
10 Aug	F62	N59°20.01	E023°15.81	*E. affinis*	Exp 3, RNA*later*, –80°C
11 Aug	LL9	N59°42.010	E024°01.809	*E. affinis*	Exp 3
11 Aug	LL7	N59°20.010	E023°15.810	*E. affinis*	RNA*later*, –80°C

Exp 1 & 2– Experiments 1 and 2, predation experiments with *Cercopagis pengoi* preying on *Eurytemora affinis*; Exp 3– Experiment 3, grazing experiment with *E. affinis* feeding on *Rhodomonas salina*; RNA*later* – preservation with RNA*later* for genetic analysis, measurements of RNA:DNA ratio, sex- and stage-related differences in pigmentation; –80°C – frozen samples used for gut fluorescence and antioxidant capacity measurements. Note that copepods used in the experiments were collected at several stations and pooled. See Material and Methods for measurement details.

### Genetic Analyses

Using a 10% Chelex buffer, genomic DNA was extracted from pigmented and unpigmented individuals preserved in RNA*later* (25 ind. group^−1^; pooled from 6 stations; Table 1) and used for amplification of COI (∼570 bp) gene. Polymerase chain reactions (PCR) were conducted using EuF1 and EuR2 primers [28]. The products were separated in 1.5% (w/v) agarose gel with a 100-bp ladder and visualized by staining with ethidium bromide. The PCR products were purified using the QIAquick PCR Purification Kit (QIAGEN) and sequenced in both directions with an ABI 3730 PRISMH DNA Analyzer at KIGene (Karolinska Institute, Stockholm). The resulting nucleotide sequences were aligned using BioEdit software [32] checking electropherograms to ensure proper base calling. The sequences were compared to all *Eurytemora* sequences reported for the Baltic Sea (Figure S2) and all unique nucleotide sequences have been submitted to the GenBank (accession numbers JQ822115-JQ822130).

Genetic diversity was compared between the pigmentation phenotypes using estimators implemented in DnaSP version 5.10 [33], including the number of haplotypes (K), haplotype diversity (Hd, i.e., probability that two randomly chosen haplotypes are different in the sample) and nucleotide diversity (π, i.e., average number of nucleotide differences per location between two sequences), and diversity per site based on the number of segregating sites (θ). A statistical parsimony haplotype network was constructed to show genetic relatedness among haplotypes using the software package HapStar [34].

### Sex- and Age- related Differences in Pigmentation

To test whether the proportion of pigmented individuals differed between males and females (CIV-VI), 25 individuals of each sex per sample were counted in the 6 field samples preserved with RNA*later* and the pigmentation status and developmental stage were noted for each individual. When no sex-related difference was found, we examined the variation between the immature (CIV-V) and mature (CVI) individuals using the same counts.

### Predation Experiments

We conducted two predation experiments using parthenogenic females of *Cercopagis pengoi* (instars II and III) as predators and older copepodites (CIV–VI, males and females without egg sacks) of *Eurytemora affinis* as a prey. In Experiment 1, predation rates (PR) of *C. pengoi* offered either pigmented or unpigmented copepods were determined, whereas in Experiment 2, prey selection using a mixed (1∶1) assemblage of pigmented and unpigmented copepods was determined. The experiments consisted of 6 replicate incubations for each treatment and were conducted in either 0.1 (Experiment 1) or 0.2 (Experiment 2) L vials under constant illumination with green light at 15°C and lasted ca. 20–22 h. Incubations were started by adding the known number of prey (10 in Experiment 1or 20 in Experiment 2 to keep the same prey concentration in both experiments) to a single *C. pengoi*. In each experiment, there were two predator-free control incubations with the same number of prey to correct for prey recovery (100% in all cases). Recovered prey were counted directly upon the experiment termination and the pigmentation status of each copepod was noted. Body length (BL, from the top of the head to the base of the caudal claws) of every *C. pengoi* and prosome length (PL) in a subsample of 20–25 individuals for each *E. affinis* group were measured under a dissecting microscope. PR was calculated as the difference between the initial and final numbers of prey and scaled to 24 h.

### Grazing Experiment

Experiment 3 was conducted to test for differences in grazing, metabolic activity and antioxidant capacity between the copepods with contrasting pigmentation. As food, we used *Rhodomonas salina* (Cryptophyceae; CCMP 1319) grown on f/2 medium at 17°C and 8‰ salinity in artificial seawater (Instant Ocean™, Aquarium Systems). To minimize algal growth during the experiment and increase accuracy of the grazing rate (GR) estimates, the algae were pre-treated with γ-radiation to prevent cell division [35]. Groups of pigmented and unpigmented copepods (CIV–VI, males and females without egg sacks; 10 ind. vial^−1^; 6 replicates per treatment) were incubated during 22–24 h in 0.2 L of FSW with 600 µgC L^−1^ of food; light regime was the same as in the predation experiments. This food concentration is above the saturation level for *E. affinis* [36] that ensures no decrease in the feeding rate due to prey depletion. To determine algal concentrations (cells mL^–1^), 15 mL of the media were preserved with acid Lugol’s solution and analyzed using a laser particle counter Spectrex PC-2000 (Spectrex Corp.). The individual GR was determined as a difference between the initial and final algal concentrations in each vial and scaled to 24 h and individual copepod.

Upon termination of the experiment, the copepods were examined with a dissecting microscope, and dead individuals were noted. In all incubations, survivorship was ≥95%, with no significant difference between the pigmented and unpigmented copepods (Kruskal-Wallis statistic = 0.91, p>0.93). Live copepods recovered from each vial were split in two groups: (1) 5 individuals designated for RNA:DNA ratio assay were transferred to Eppendorf tubes containing 100 µL of RNA*later* and stored at 4°C [37], and (2) 4–5 individuals designated for antioxidant capacity measurements were transferred to Eppendorf tubes and stored at –80°C.

### Gut Fluorescence

Using the frozen zooplankton samples collected in the field, gut pigment concentration (GPC) in *Eurytemora affinis* (females, CV-VI) was analyzed fluorometrically. After thawing on ice, the copepods were quickly sorted in groups of 5 individuals per pigmentation type; 2 replicate samples per station, 5 stations in total (Table 1). Gut pigments were extracted in 0.5 mL of 90% acetone for 24 h at 4°C in the dark. After centrifugation for 3 min at 3000 rpm, the fluorescence was measured using a Turner Designs TD700 fluorometer with a detection limit of 0.06 µg L^–1^ of chlorophyll-a (Chl-a) and 0.08 µg L^–1^ of phaeophytin-a. Fluorescence was measured before and after acidification with HCl and GPC, and Chl-a was calculated as ng Chl-a eq ind^ –1^ [38]. The duplicate samples were averaged to provide a single value per station for each pigmentation type.

### RNA:DNA Ratio

This ratio was used as a proxy for overall metabolic activity in the copepods [39]. The nucleic acid concentrations were measured in both field samples preserved in RNA*later* (5 stations, LL6 excluded; Table 1) and those collected upon termination of the grazing experiment (6 replicates). For each replicate, a pooled sample of 5 individuals was used to quantify RNA and DNA concentrations (µg ind.^−1^) using microplate fluorometric high-range RiboGreen (Molecular Probes, Inc. Eugene, OR) assay [40] optimized for *Eurytemora affinis* of similar size [41]. Test samples, standards, and the negative controls were measured in duplicates using FLUOstar Optima microplate reader in black solid flat-bottom microplates (Greiner Bio-One GmbH) at excitation/emission wavelengths of 485/590 nm (0.2 s well^−1^, 10 measurements well^−1^). The DNA:RNA standard curve slope ratio was 1.76.

### Total Antioxidant Capacity

These measurements were done using copepod samples obtained in the Experiment 3 (6 replicates) and frozen samples of field-collected copepods that were also used for the GPC (5 stations; Table 1). The total antioxidant capacity was measured as oxygen radical absorbance capacity (ORAC), with fluorescein as a fluorescent probe, AAPH (2,2-azobis(2-amidinopropane) dihydrochloride as a thermal free radical source and Trolox as a standard [42]; all reagents were purchased from Sigma-Aldrich. The copepods (4–5 individuals sample^−1^) were homogenized in Tris buffer with ∼50 µg of glass beads (<100 µm) for 2 min at 4°C using FastPrep, centrifuged at 15000×g for 15 min at 4°C and then 10 and 30 µL of the supernatant were taken for measurements of ORAC and protein content, respectively. Protein concentration (mg mL^−1^) was measured using the bicinchoninic acid assay (BCA, Pierce Ltd.) according to the manufacturer’s instructions. The ORAC values were expressed in Trolox-equivalents, µg mg protein^−1^. All measurements were conducted in duplicates that were averaged for statistical analysis.

### Egg Ratio

Egg ratio was calculated using the field samples preserved in RNA*later* (6 stations; Table 1). In each sample, the ratio was calculated using ∼50 females for pigmented and unpigmented *E. affinis* and dividing a cumulative number of eggs by a total number of mature females in each category.

### Data Analysis and Statistics

To assess sex- and age-related differences in pigmentation occurrence, Wilcoxon matched-pairs signed-ranks test was used. Differences in PR (Experiment 1) and GR (Experiment 3) between the pigmentation types were evaluated by an unpaired t-test. To test for differences in prey preference, Manly’s α index [43] was calculated for each prey type in the Experiment 2. This estimator has been shown to be asymptotically distributed as a normal random variate [43]; therefore to assess effects of pigmentation on PR and prey preference, we compared PR observed in the mixed prey incubations and mean α values for pigmented and unpigmented prey using a paired t-test.

Pearson’s product-moment and correlation coefficients were calculated for all pairs of measured physiological and biochemical variables. To further evaluate effect of pigmentation on RNA:DNA ratio and ORAC, 2-way ANOVA was used with pigmentation status (pigmented or unpigmented) and animal origin (field or experiment) as categorical predictors. To compare egg ratio and GPC between the pigmentation types, paired t-test was used with pairing by station. Finally, to establish a relationship between the metabolic status and antioxidant capacity and to evaluate effect of pigmentation on this relationship, we used a general linear model (GLM) with the pigmentation status and the animal origin as the categorical variables and ORAC as continuous variables and RNA:DNA ratio as the dependent variable. The data for the correlation and regression analyses were Box-Cox transformed to improve distribution of the residuals. If not specified otherwise, data are presented as means and standard deviations (SD). Statistica version 8.0 (StatSoft, 2007) was used for statistical analyses; the differences were considered significant at p<0.05.

## Results

### Species Identity and COI Variability in *E. affinis*

All 50 sequences had high identity (98–100%) to COI sequences reported for the Baltic lineage of *Eurytemora affinis* from coastal areas of the Gulf of Finland (e.g., Vyborg Bay: HM473964- HM473975, HM473980- HM473982; Luga Bay: HM473994, HM473996- HM473998), but also from other parts of the Baltic Sea (e.g., Swedish coastal waters: JF727517- JF727523; Gulf of Riga: HM474023, HM474026- HM474027); see Figure S2. By contrast, there were only 87–88% identity to the sequences representing *E. carolleeae* of North American origin (Luga Bay: HM474011- HM474012, HM474003; Neva Bay: HM474029). Therefore, all species in our collections were assumed to belong to the Baltic lineage of *E. affinis*.

**Figure 1:**
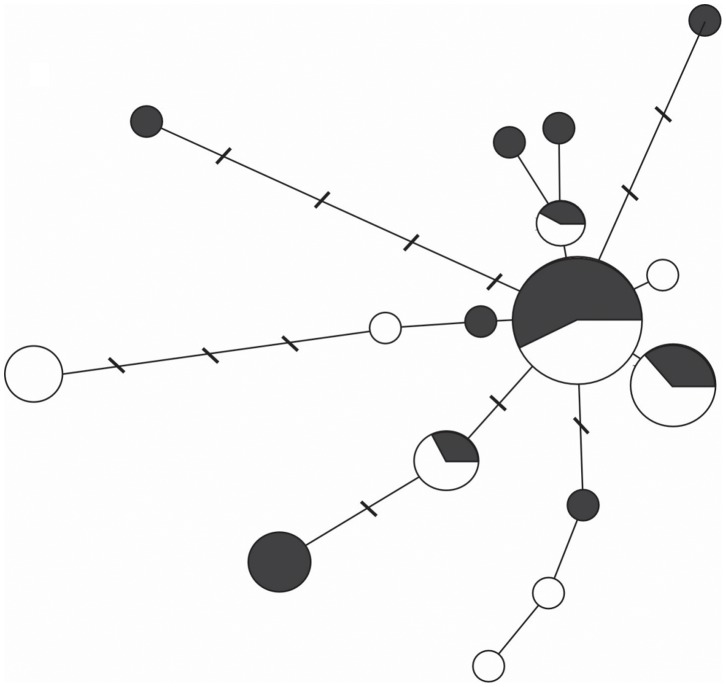
Minimum spanning tree showing relationships among haplotypes of pigmented (black) and unpigmented (white) *Eurytemora affinis*. Size of circle is proportional to the frequency of the haplotype. Slash marks indicate the number of nucleotide substitutions between haplotypes; no slash marks present indicates a single substitution separating haplotypes.

The aligned COI sequence dataset contained 16 unique haplotypes with 20 polymorphic sites sampled across 571–574 bp. In both pigmentation groups, many closely related haplotypes co-existed, with high haplotype diversity and low nucleotide diversity (Table 2). Four haplotypes were shared between the groups, whereas 7 and 5 haplotypes were private for the pigmented and unpigmented copepods, respectively (Fig. 1). There were two prevalent haplotypes, while rare haplotypes were in general one to three mutational steps apart from the prevalent haplotypes.

**Table 2:** Eurytemora affinis.

Pigmentation type	K	Hd	π	θ
Pigmented	11	0.860	0.0042	0.0074
Unpigmented	9	0.863	0.0039	0.0051
Total	16	0.859	0.0041	0.0077

Measures of genetic diversity of the cytochrome *c* oxidase subunit I (COI) in the copepods with different pigmentation type.

Number of haplotypes (K), haplotype diversity (Hd) and nucleotide diversity (π), and diversity per site (θ).

### Sex- and Age- related Differences in Pigmentation

Neither sex- (Wilcoxon matched-pairs signed-ranks test: p>0.8) nor age-related (p>0.5) differences in pigmentation were significant. The proportion of the pigmented individuals varied from 25% to 42% among the stations.

### Predation on Pigmented and Unpigmented *Eurytemora*

**Figure 2:**
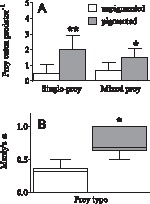
Predation rate and prey preference by *Cercopagis pengoi* offered pigmented and unpigmented *Eurytemora affinis* in feeding trials (Experiments 1 and 2). (A) predation rate (PR) in single-prey incubations (Experiment 1; mean ±SD; n = 12), and (B) prey preference index (Manley’s α) estimated from the mixed-prey incubations (Experiment 2; Tukey’s box and whisker plot, n = 12); the index ranges from 0 to 1, with higher values indicating greater preference. Shown are the median value (horizontal line), 25% to 75% response ranges (top and bottom lines of boxes) and minima and maxima (whiskers). Asterisk indicate a significant difference (p<0.05) in preference for the pigmented copepods when compared to those without visible pigmentation.

In the single prey type experiment, the effect of pigmentation on PR was significant (unpaired t-test; t_10_ = 3.503, p<0.006; Fig. 2A), with the pigmented copepods being consumed at ∼4-fold higher rate than unpigmented ones. Similarly, in the mixed prey experiment, the effect of pigmentation on PR was significant (paired t-test; t_5_ = 5.000, p<0.003; Fig. 2A), with the pigmented copepods being consumed at ∼2-fold higher rate than unpigmented ones. This was reflected by the significantly higher Manly’s α for the pigmented individuals in the mixed prey incubations (paired t-test; t_10_ = 2.72, p<0.03; Fig. 2B) indicating that pigmentation increases vulnerability to predation. The observed differences were not related to body size variations in either the predator (*C. pengoi* BL: t_48_ = 0.97, p>0.43) or the prey (copepod PL: t_48_ = 1.01, p>0.32).

### Copepod Grazing Rate in the Experiment and Gut Fluorescence *in situ*

**Figure 3:**
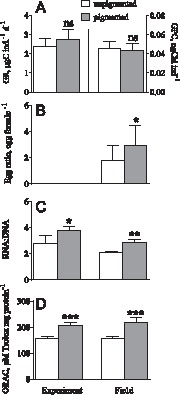
Feeding, growth, reproduction and antioxidant capacity in pigmented and unpigmented *Eurytemora affinis* in field and experimental studies. (A) grazing rate (GR; left axis, n = 12) on *Rhodomonas salina* and gut content fluorescence (GCF; right axis, n = 10) measured in the field collected copepods; (B) egg ratio in the samples collected in the study area (n = 12); (C) RNA:DNA ratio in the copepods (Experiment 3: n = 12; field samples: n = 10), and (D) total antioxidant capacity assayed as ORAC (Experiment 3: n = 12; field samples: n = 10). All data are shown as mean ±SD.

There was no significant difference in GR between the pigmented and unpigmented copepods fed *Rhodomonas* in Experiment 3 (unpaired t-test: t_10_ = 1.40, p>0.19; Fig. 3A). Similarly, GPC in field collected individuals showed no significant difference between the pigmentation types (paired t-test: t_4_ = 0.78, p>0.49; Fig. 3A), with mean values varying from 0.02 to 0.07 ng Chl-a eq ind^ –1^.

### Egg Ratio, Metabolic State and Total Antioxidant Capacity

Pigmentation had significantly positive effects on egg ratio in the field-collected *E. affinis* (paired t-test: t_5_ = 3.91, p<0.02; Fig. 3B). Similarly, there were significantly higher values for RNA:DNA ratio (2-way ANOVA; F_1,20_ = 16.39, p<0.007; Fig. 3C) and ORAC values (F_1,18_ = 107.3, p<0.0001; Fig. 3D) in the pigmented copepods. Moreover, the experimental conditions with *ad libitum* feeding affected positively the RNA:DNA ratio compared to the field-collected copepods (F_1,20_ = 14.02, p<0.002), whereas no significant differences in ORAC values between the animals used in the experiment and those in the field samples were observed (F_1,18_ = 1.505, p>0.3). In no case the interaction term was significant (p<0.4 in all cases).

There were significant positive correlations between ORAC and growth indices, but not with food intake (Table 3). Finally, there was a significant relationship between the RNA:DNA ratio and ORAC (GLM; F_1,19_ = 4.98, p<0.04), with a slope being significantly higher in animals used in Experiment 3 compared to the field samples (F_1,19_ = 15.33, p<0.002), whereas pigmentation did not affect the relationship (F_1,19_ = 0.93, p>0.8).

**Table 3:** Eurytemora affinis.

Variable	RNA:DNA	Egg ratio	GR	GCF
ORAC	**0.59** (22)	**0.63** (10)	0.44 (12)	0.53 (10)
RNA:DNA		0.61 (10)	0.46 (12)	0.51 (10)
Egg ratio				0.44 (10)

Correlation matrix with Pearson correlation coefficients among the total antioxidant capacity (ORAC), growth indices (RNA:DNA ratio and egg ratio), and food intake (GR and GCF).

ORAC: oxygen radical absorbance capacity (Experiment 3 and field collected copepods), GR: grazing rate (Experiment 3), egg ratio: number of eggs per female in a sample (field collected copepods), GCF: gut content fluorescence (field collected copepods). Significant correlations (p<0.05) are in bold face. Data were Box-Cox transformed to approach normal distribution. Number of samples is shown in parentheses.

## Discussion

In *Eurytemora affinis*, pigmentation carries both penalty in terms of the greater vulnerability to predation and fitness advantage in terms of enhanced growth, reproduction and metabolism. Three crucial results support this conclusion. First, higher predation rates and positive selectivity for the pigmented copepods were observed in the predation experiments with the visual predator *Cercopagis pengoi*. Second, growth-related variables (egg ratio and RNA:DNA ratio) were consistently higher in the pigmented copepods, whereas the food intake was similar between the pigmentation types. Consequently, the higher growth output in the pigmented individuals at a given feeding rate implies higher growth efficiency, the capacity to convert ingested energy into biomass. Third, the pigmented copepods had higher antioxidant capacity, suggesting that this phenotype is superior in handling excessive formation of free-radicals, which is often a primary cause or a downstream consequence of tissue damage due to various environmental stressors, including predation pressure [44]. These findings have important implications for food web interactions as increased contribution of the pigmented forms in the population would have positive effects on energy transfer from primary producers to secondary consumers and prey quality, in terms of its antioxidant content, for zooplanktivores.

Our results that pigmented individuals have higher egg production, metabolic activity and antioxidant capacity lend support to the metabolic stimulation hypothesis [12]. Despite experimental evidence that pigmentation enhances survival of zooplankton exposed to high irradiances, field studies often show lack of significant relationships with irradiance, but strong inverse relationships with temperature [6], [12]. In *Eurytemora affinis*, juvenile development is highly temperature dependent in the range of 7 to 18°C [45], i.e., common summer temperatures in the trophogenic zone in the Baltic Sea. Therefore, increased metabolic stimulation through absorption of solar radiation would translate into increased individual and population growth. More recently, a field study reporting lack of a direct relationship between levels of photoprotective compounds in freshwater copepods and water column radiation provided further support to Byron’s hypothesis [46]. The metabolic stimulation hypothesis was, however, criticized, arguing that the possible temperature gain is insignificant due to the high heat transfer in small-sized planktonic animals [47].

We suggest an alternative mechanism for the metabolic stimulation that could involve elevated antioxidant levels as a primary means of increasing metabolism without temperature increase. As physiological functions of pigments include antioxidant protection, the observed higher total antioxidant capacity (Fig. 3D) in the pigmented copepods is likely to simply reflect higher concentration of pigments contributing to the higher antioxidant defences in this phenotype [48]. Recently, a number of studies have attempted to reveal the relationships between antioxidant capacity and fitness components in various animals [20]. Although the connections appear to be stage- and species-specific, higher antioxidant levels were linked with larger clutches [49], greater reproductive success [50], improved offspring quality [51], and increased life span [52]. The observed positive associations between ORAC levels and growth-related variables (reproductive output and RNA:DNA ratio) with no apparent correlation to the food intake (Table 3) provides further evidence for the positive effects of antioxidant capacity on the copepod fitness. Moreover, the relationship between ORAC and RNA:DNA ratio was not phenotype-specific, indicating common underlying mechanisms.

The apparent lack of costs for pigment synthesis is in disagreement with experimental studies on zooplankton inhabiting high-UVR lakes and suffering growth penalties related to production of photoprotective compounds [4], [23]. In our study, the *in situ* growth conditions for copepods were suboptimal as indicated by the higher RNA:DNA ratio of the copepods incubated at *ad libitum* food levels in the grazing experiment compared to the field-collected copepods (Fig. 3B); hence, it is difficult to assess costs because each phenotype is likely to face own challenges. Moreover, our study design might have failed to detect an existing cost: we measured feeding either in the surplus of high quality food in the absence of competition or external stressors (Experiment 3) or using a gut fluorescence (field-collected copepods, pooled samples) without any knowledge on the gut evacuation governed by ambient temperature and food levels. Fitness trade-offs can be obscured in the presence of abundant resources, whereas ambient conditions may differ in multiple variables, such as temperature, food quality and quantity, particularly if the pigmentation phenotypes resided at different depths.

Our study was not designed to address UVR effects on pigmentation in *Eurytemora affinis*, because it seemed unlikely that these effects would be ecologically relevant in the system studied. Indeed, detrimental UVR effects on copepods were found to be restricted to the first meter of the water column [53], particularly in sea areas with high primary productivity. The actual levels of UVR that summer zooplankton communities in the Baltic Sea are exposed to are relatively low compared to the oligotrophic and/or high elevation shallow lakes in Scandinavia, with high light penetration and a lack of depth refuge from UVB exposure for zooplankton. In the Baltic Sea, plankton communities are more protected from UV exposure, because of the greater depth and high concentrations of colored dissolved organic matter contributing to high attenuation [53]. In the coastal areas, a typical 1% penetration depth for UV-B is ∼0.1 m in inner bays and 0.3 m in outer areas, whereas near infrared wavelength range that contributes most to the temperature-mediated metabolic stimulation reaches deepest in the water column [54]. Moreover, our study was conducted during a filamentous cyanobacteria bloom, which substantially increases water turbidity (Secchi depth <4.5 m) and thus decreases UVR penetration in the water column. Recently, a counter-intuitive contrast in pigmentation of the copepod *Arctodiaptomus spinosus* in shallow fishless lakes has been reported, with unpigmented copepods inhabiting macrophyte-dominated clear water lakes and pigmented forms occurring in highly turbid and phytoplankton-rich lakes [55]. While the penetration of UV radiation alone failed to explain the differences in copepod pigmentation among these lakes, several possible stressors, such as wind-induced turbulence combined with short-term sunlight exposure and crowding, were advocated in explaining this pattern [55]. In particular, the pigment accumulation in the crowded populations occurring in the turbid lakes was suggested to be a support for immune defense in copepods [55], [56]. Thus, high UVR exposure is not the only factor favoring carotenoid accumulation in zooplankton. Nevertheless, the UVR effects on the copepod pigmentation in the Baltic Sea cannot be ruled out completely. It could be hypothesized, for example, that unpigmented phenotype (∼70% of the population) would be more sensitive to UVR avoiding illuminated waters during daytime, whereas pigmented individuals (∼30%) would stay in the more productive upper part of the water column and produce more offspring – all these at the cost of predation risk. In this context, examining the vertical distribution of copepods with contrasting pigmentation pattern, would help in (1) understanding proximate causes of DVM, (2) establishing linkages between pigment production and vertical position of the copepods in the water column, and (3) revealing energetic costs related to migratory activity, which would allow to compare growth efficiencies between the phenotypes and to evaluate the trade-offs involved.

Whereas defence against predators is usually accompanied by declining rates of growth or development in prey organisms, the underlying physiological responses remain little understood and often explained by reduced feeding and/or increased metabolic costs due to the fight-or-flight response [57]. In our case, the lower egg ratio in the unpigmented *E. affinis* cannot be explained by reduced foraging under predation risk as indicated by similar gut pigment content. Instead, we suggest that when predation pressure is high, the unpigmented copepods have higher survival rate, but also lower antioxidant capacity and reduced fitness. In line with this, predation-induced decrease in antioxidants has been observed in copepods [56] and damselfly [44], resulting in a weakened immune status and a growth reduction, respectively. It remains to be studied, whether each individual adjust the pigmentation and related antioxidant reserves to the prevailing predation risk according to its individual costs and benefits or, alternatively, whether copepods with different pigmentation suffer differential mortality as a result of selective predation on specific genotypes.

Although, mitochondrial polymorphisms have been found to be linked with pigmentation patterns in various species (e.g., cladocerans [24], shrimps [25], insects [58], fish [59]), there was no association between pigmentation and mtDNA genotypes in the Baltic *Eurytemora affinis*. This does not, however, imply an absence of genetic differences between the copepods with different pigmentation phenotypes, both with regard to genes responsible for positioning pigments in space and time and those responsible for synthesis of pigments. Recently, the predation and photoprotection concepts have been integrated [60], demonstrating that zooplankters are plastic in their pigmentation, making a trade-off between high and low pigmentation in relation to the prevalent fish/UV threat ratio [2], [8]. What remains to be integrated in this theory is the genetic basis of pigmentation, and this calls for a new approach to study adaptive variation in production of various pigments and their relative importance in different species and systems. Indeed, in some cases, pigmentation polymorphism could be genetically determined, in others, it is a polyphenism, a developmental difference in pigmentation pattern cued by physical or chemical signals. With regard to dietary pigments, such as carotenoids and mycosporine-like amino acids, the latter has been found to be the case [5], [56]. However, both production of the cuticular pigments and pigment-dispersing hormone activities are under genetic control [61], [62] and, therefore, *E. affinis* pigmentation could be an example of a genetic system that leads to multiple fitness peaks under multiple selection pressures.

In this respect, *E. affinis* could represent a particularly suitable model, due to its occurrence worldwide and high evolutionary diversification [28], [29], [63]. Together, the observed low nucleotide-diversity and high haplotype-diversity indicate a co-existence of a high number of closely related haplotypes (Fig. 1), which is indicative of a recent population expansion. These findings are in agreement with the results of a pairwise haplotype mismatch analysis [28] suggesting that Baltic *E. affinis* has undergone a recent expansion. High genetic diversity in such populations is the raw material for natural selection, and therefore, potentially underpins fitness and/or adaptive capacity. Recently, a compelling evidence has been presented on the heritable genetic variation of pigmentation as a fitness-related trait in populations of snakes [64] and isopods [65]. Given the involvement of pigmentation in fitness traits of *E. affinis* and a likely selection by predation, understanding genetic basis of pigmentation in this species may provide a more comprehensive view on ecological and evolutionary significance of pigmentation patterns in copepods from temperate estuarine systems, which might be different from those in clear lakes exposed to high UVR levels.

## Supporting Information

Figure S1 **A schematic drawing showing pigmentation pattern in pigmented (A) and unpigmented (B)** ***Eurytemora affinis*** **.**
(PDF)

Figure S2 **Maximum Likelihood (ML) tree for** ***Eurytemora affinis*** **sequences reported from the Baltic Sea.**
(PDF)
