# Supplementary material for: Trade-Offs between Predation Risk and Growth Benefits in the Copepod Eurytemora affinis with Contrasting Pigmentation
Source: PLoS One. 2013 Aug 7;8(8):e71385. doi: 10.1371/journal.pone.0071385 (PMC3737102; doi:10.1371/journal.pone.0071385)

Supporting Information

Trade-offs between predation risk and growth benefits in the copepod *Eurytemora affinis* with contrasting pigmentation

By Elena Gorokhova, Maiju Lehtiniemi, and Nisha H. Motwani

Figure S1. A schematic drawing showing pigmentation pattern in pigmented (A) and unpigmented (B) *Eurytemora affinis*.

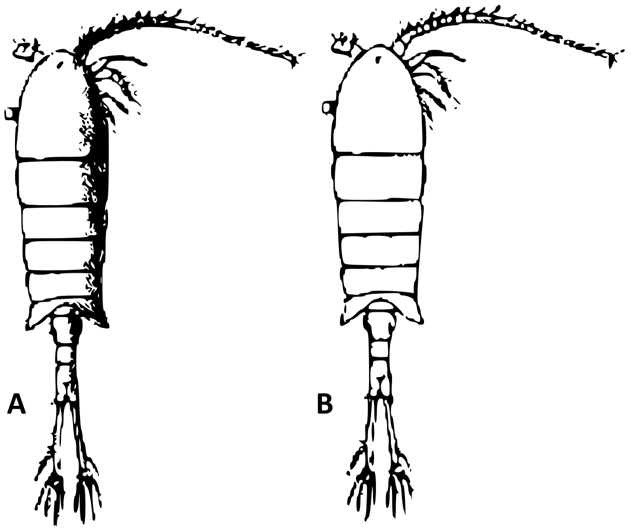

Supplement: Figure S1 — A schematic drawing showing pigmentation pattern in pigmented (A) and unpigmented (B) Eurytemora affinis . (PDF) [file pone.0071385.s001.pdf]
