# Supplementary material for: Trade-Offs between Predation Risk and Growth Benefits in the Copepod Eurytemora affinis with Contrasting Pigmentation
Source: PLoS One. 2013 Aug 7;8(8):e71385. doi: 10.1371/journal.pone.0071385 (PMC3737102; doi:10.1371/journal.pone.0071385)

Supporting Information

Figure S2. Maximum Likelihood (ML) tree for *Eurytemora affinis* sequences reported from the Baltic Sea.

Sequences are presented with their GenBank accession numbers preceded by geographic locations that are coded as follows:

Askö: Swedish coastal area close to Askö station, northern Baltic proper;
Elba: Elba estuary, southern Baltic Sea
Himm: Himmerfjärden Bay, Swedish coastal area in Södertälje municipality, northern Baltic proper;
Luga: Luga Bay, Gulf of Finland;
Riga: Riga Bay, northern Baltic proper;
Vist: Vistula Lagoon, south-east Baltic proper;
Vybo: Vyborg Bay, Gulf of Finland.

Sequences marked **U** and **P** are unpigmented and pigmented copepods, respectively, sequenced in this study, the number indicates isolate.

Phylogenetic tree was constructed with the software MEGA 5.0. Identical sequences were not included in the tree. One thousand bootstrapped replicate resampled datasets were analyzed. Sequences representing *Eurytemora carollee* are marked in red. *Eurytemora lacustris* was used as an outgroup.

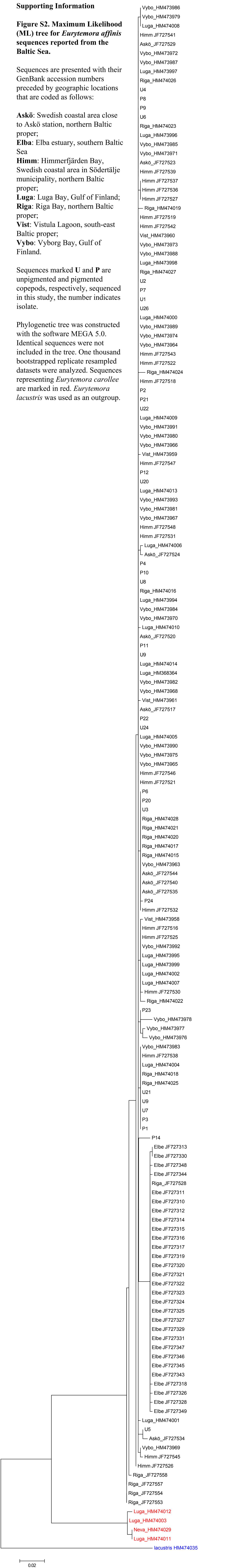

Supplement: Figure S2 — Maximum Likelihood (ML) tree for Eurytemora affinis sequences reported from the Baltic Sea. (PDF) [file pone.0071385.s002.pdf]
